# Effect of Deworming on Health Outcomes among Children Aged 12–59 Months in Tanzania: A Multilevel Mixed Effects Analysis

**DOI:** 10.1155/2023/9529600

**Published:** 2023-07-21

**Authors:** Cypriana Cyprian Moshi, Penina Joseph Sebastian, Kaunara Ally Azizi, Erick Killel, Devotha Gabriel Mushumbusi, Wessy Pirbhai Meghji, Malimi Emmanuel Kitunda, Francis Karl Millinga, Hancy Adam, Ladislaus Manaku Kasankala

**Affiliations:** Tanzania Food and Nutrition Center, P.O. Box 977, Dar es Salaam, Tanzania

## Abstract

**Introduction:**

Mass deworming of preschool children is a strategy suggested to prevent soil-transmitted helminth infections in most developing countries. Nonetheless, there is a scarcity of data showing the contribution of mass deworming to a child's nutritional status. The purpose of this study was to assess the effect of deworming on nutritional health outcomes (stunting, underweight, and anemia) in children aged 12 to 59 months.

**Methods:**

A secondary analysis of data extracted from the Tanzania Demographic and Health Survey (TDHS) 2015-16 data was carried out. A total of 7,962 children were included in this study. A multilevel logistic regression was used at a 5% level of significance to determine the individual- and community-level determinants of deworming on health outcomes among children.

**Results:**

The prevalence of underweight (62.6%), stunting (61.0%), and anemia (61.8%) was higher in children who were not dewormed than those who were dewormed. Female children were more likely to suffer from poor health outcomes (OR = 1.01 and 95% CI = 0.95–1.07) than male children. Children aged 24–35 months and 36–47 months were significantly less likely to suffer from poor health outcomes (OR = 0.89; 95% CI = 0.82–0.97 and OR = 0.88; 96% CI = 0.81–0.96, respectively; *p* < 0.01). Children from households with unimproved toilets (OR = 1.38 and 95% CI = 1.25–1.52), unimproved water sources (OR = 1.08 and 95% CI = 1.01–1.16), and living in rural areas (OR = 1.02 and 95% CI = 0.91–1.14) had higher odds for poor health outcomes.

**Conclusion:**

Deworming may be an effective technique for preventing poor health outcomes in children and the risks associated with them, such as poor growth and development.

## 1. Introduction

More than one billion individuals in developing countries are infected with soil-transmitted helminths (STH), with roughly one-third of the world's population afflicted with at least one STH species [1, 2]. The World Health Organization (WHO) [3] estimates that 270 million preschool children in developing countries are infested with species such as whipworms (*Trichuris trichiura*), roundworms (*Ascaris lumbricoides*), and hookworms (*Necator americanus* and *Ancylostoma duodenale*). Heavy worm infestation has been associated with malnutrition, in particular stunting, underweight, and anemia in children [4–6].

STHs have varied nutritional outcomes to the host as they compete for micronutrient absorption in the small intestine and feed on host tissues such as blood and serum, causing iron and protein loss. They also cause maldigestion or malabsorption of nutrients. Moreover, feeding on the contents in the gut including the host's secretions that make up the exoenteric circulation decreases appetite, lowering the food intake of an infected individual [7, 8].

Physical growth and cognitive development are significantly affected when children are chronically infected with STH [9]. Despite other factors associated with anemia, such as diet, infections, and genetics, the prevalence of anemia in developing countries has been greatly contributed by intestinal parasite infections among children [10]. Several studies conducted in different parts of the world also showed a strong association between STH and iron deficiency anemia in children [9, 11, 12].

To lessen the burden of worm infestation in the community, WHO recommends all preschool children and other risk groups receive periodic pill therapy (deworming) once or twice a year, with the most common being albendazole 400 mg and mebendazole 500 mg. In locations where the prevalence of infection is greater than 20%, deworming is recommended once a year, and twice a year in areas where the incidence is greater than 50% [3]. The goal of the deworming program is to reduce the number of children with moderate or high-intensity infections, which are associated with poor health outcomes.

Tanzania, like other underdeveloped nations, has a high prevalence of STH parasite transmission; hence, children between the ages of 12 and 59 months undergo deworming twice a year. However, there is a scarcity of data on the effects of the deworming campaigns on the nutritional health outcomes of the targeted age group. Therefore, the purpose of this study was to assess the effect of deworming on nutritional health outcomes in children aged 12 to 59 months. The study's findings will be used by government ministries, policy makers, and development partners to improve deworming interventions in the country.

## 2. Methodology

### 2.1. Study Design

**Figure 1:**
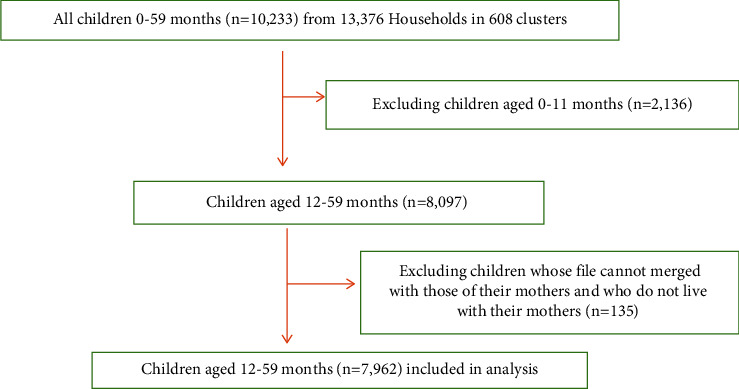
Flowchart showing selection of children aged 12 to 59 months from TDHS 2015 to 16.

**Figure 2:**
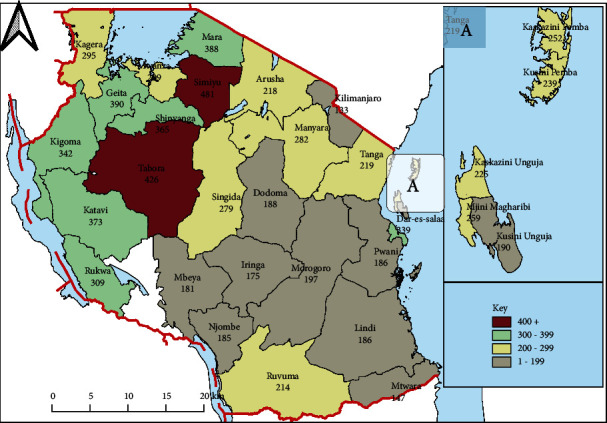
Number of the samples from each region collected in this study.

A secondary analysis of data extracted from the Tanzania Demographic and Health Survey (TDHS) 2015-16 data was carried out. The study methodology is described in detail in the TDHS 2015-2016 published report [13]. In summary, the TDHS used a multistage cluster sampling design in which 608 clusters were selected at the first stage and 22 households from each selected cluster were sampled at the second stage. The survey included 10,233 children aged 0–59 months from all regions of Tanzania who were matched with their mothers. Only 7,962 children aged from 12 to 59 months were chosen for this study. A detailed description of the procedure used to select children is given in Figure 1. The number of samples from each region collected for this study is depicted in Figure 2.

### 2.2. Outcome Variables

Health outcomes variables (stunting, underweight, and anemia) were the three dependent variables investigated in this study. Based on WHO guidelines, a child is stunted if his or her height for age *Z*-score (HAZ) is less than −2 SD, and underweight if his or her weight for age *Z*-score (WAZ) is less than −2 SD. To create a dichotomous variable, stunted or underweight children were assigned 1 and assigned 0 if they were normal. Anemia is defined as a deficiency of hemoglobin (Hb) to provide adequate oxygen to bodily tissues. According to the WHO, a child has anemia if his or her Hb level is less than 11 grams per deciliter (g/dL). Informing dichotomous variables, being anemic was assigned 1 and 0 for nonanemic (normal). Households were given scores based on the number and kinds of consumer goods they owned, ranging from a television to a bicycle or car, plus housing characteristics, such as the source of drinking water, toilet facilities, and flooring materials. These scores were derived using principal component analysis. The wealth index variable categorizes the household's wealth status into three classes: poor for the poorest and poor, normal (neutral) for the middle, and rich for the richer and richest.

### 2.3. Explanatory Variables

Eight explanatory variables were considered in this study based on their association with deworming status. These variables were classified into two groups: individual-level variables and community-level variables.

#### 2.3.1. Individual-Level Variables

The child's age (month), sex of child, mother's education level, and mother's age, and type of toilet facility were included as individual-level variables.

#### 2.3.2. Community-Level Variables

Wealth quantile, type of place of residence (urban and rural), and water source were regarded as community-level variable.

### 2.4. Data Analysis

The STATA software, Version 15 (StataCorp LLC, USA), was used for analysis. As part of this analysis, all data were cleaned, processed, and recorded in the appropriate format. The characteristics of the study population were described using frequencies in the descriptive analysis. A multilevel logistic regression was used at a 5% level of significance to determine the effect of individual- and community-level determinants of deworming on three health outcomes (stunting, underweight, and anemia) among children. The *p* value <0.05 was considered statistically significant. Descriptive statistics were displayed for individual and community variables. Three models are displayed in this analysis: model I (contains individual variables), model II (contains community variables), and model III (both individual and community variables).

## 3. Results

### 3.1. Description of Social-Demographic Characteristics of Study Participants in Association with Deworming

The total weighted samples of 7,962 children ranging in age from 12 to 59 months were included in this study (Table 1). Gender was represented equally, but males (42.1 percent) were dewormed more than females. Children aged 12–23 months were higher presented than other age groups (28.1 percent), but the percentage of dewormed children was nearly equal across all age groups. Children who were more dewormed came from a better toilet-equipped household, a wealthy family, had a mother who was older than 30 years, and had secondary or higher education.

### 3.2. Prevalence of Underweight, Stunting, and Anemia and Association with Deworming in Children

Overall, 15 percent of children were underweight, 39.5 percent were stunted, and 55.8 percent were anemic (Table 2). The prevalence of underweight (62.6%), stunting (61.0%), and anemia (61.8%) were higher in children who were not dewormed than those who were dewormed.

### 3.3. Multilevel Mixed Effect Logistic Regression Analysis

#### 3.3.1. Individual-Level Variables

Children aged 24–35 months and 36–47 months were significantly less likely to suffer from poor health outcomes (odds ratio (OR) = 0.89; 95% confidence interval (CI) = 0.82–0.97 and OR = 0.88; 95% CI = 0.81–0.96, respectively; *p* < 0.01). Female children were more likely to suffer from poor health outcomes (OR = 1.01 and 95% CI = 0.95–1.07) than male children. In addition, children whose mothers were aged 25–34 were significantly less likely to suffer from poor health outcomes (OR = 0.89; 95% CI = 0.81–0.97; and *p* < 0.01) than those whose mothers were aged 15–24, and children whose mothers had attended school were less likely to suffer from poor health outcomes (OR = 0.58; 95% CI = 0.49–0.68; and *p* < 0.01) than those whose mothers had not. Lastly, children from households with unimproved toilets were more likely to suffer from poor health outcomes (OR = 1.38; 95% CI = 1.25–1.52; and *p* < 0.01) than those using improved toilets (Table 3).

#### 3.3.2. Community-Level Variables

Children from rich families were significantly more likely protected from poor health outcomes (OR = 0.69; 95% CI = 0.62–0.76; and *p* < 0.01), and compared with urban children, rural children had higher odds for poor health outcomes (OR = 1.02 and 95% CI = 0.91–1.14). Lastly, children from households with unimproved water sources were more likely to suffer from poor health outcomes (OR = 1.08; 95% CI = 1.01–1.16; and *p* < 0.05) than those using improved water sources (Table 3).

#### 3.3.3. Individual- and Community-Level Variables

Model III included both individual- and community-level variables. The findings of model III show that an increase in maternal education level, specifically higher education, reduces the chances of their children having poor health outcomes (OR = 0.61; 95% CI = 0.52–0.72; and *p* < 0.01). The results also indicate that children from households with unimproved toilets have a higher risk of poor health outcomes (OR = 1.18; 95% CI = 1.02–1.37; and *p* < 0.05). Conversely, children from wealthier households are less likely to experience poor health outcomes (OR = 0.81; 95% CI = 0.70–0.93; and *p* < 0.01). In addition, children from households with unimproved water sources are marginally more likely to suffer from poor health outcomes (OR = 1.01 and 95% CI = 0.92–1.11) (Table 3).

## 4. Discussion

This study found a significant association between deworming treatment and anemia among children aged 12 to 59 months who were involved in the study. However, there was no significant association between deworming treatment and being underweight or stunted.

This study found a lower likelihood of anemia in dewormed children compared to their counterparts. Studies have found that children who received deworming treatment had significantly lower levels of anemia than those who did not [14–16]. This is likely due to the fact that deworming can reduce the amount of nutrients lost to parasitic infections, allowing more nutrients to be available for hemoglobin synthesis, and other processes involved in the production of healthy red blood cells. In addition, deworming can reduce the risk of infection and inflammation, which can also contribute to anemia [17]. Our finding compares well with previous studies conducted in Zanzibar, Tanzania, and Thailand, which found significant improvements in hemoglobin concentration and a decrease in anemia prevalence in children after deworming treatment [18, 19]. Such a dramatic change in the anemic condition after deworming treatment strongly suggests that the anemia in the study subjects was primarily caused by hookworm infection, rather than an iron deficiency caused by poor nutrition. Parasites live in a dynamic relationship with their hosts who provide all the energy and nutrients required, resulting in reduced food intake and poor nutrition of the host, causing changes in the intestinal walls, thus it causes inefficient digestion and absorption. Heavier infections can cause a range of symptoms including intestinal manifestations (diarrhea and abdominal pain), malnutrition, general malaise and weakness, and impaired growth and physical development [20]. Infections of very high intensity can cause intestinal obstruction that should be treated surgically. For example, *Necator americanus* (hookworm) have shown to reduce iron absorption, and in some instances to be associated with iron deficiency anemia [21]. Chergui and colleagues reported severe iron deficiency anemia due hookworm infection in a 34-year-old Pakistani patient with a barefoot walking habit. A barefoot walking is very common in young children in sub-Saharan countries [22]. Other studies have suggested that deworming may also reduce anemia indirectly [23, 24]. This can be seen in areas with a high prevalence of intestinal worms, where anemia is prevalent due to poor nutrition and low socioeconomic status, and not due to the direct effect of the parasites. In such areas, deworming may lead to improved nutrition, and, in turn, improved anemia rates. Thus, in addition to encouraging nutrition education and feeding practices, periodic deworming may be an effective technique for preventing anemia in children and the risks associated with it, such as fatigue, headaches, and poor growth and development.

No significant association was observed between deworming with neither stunting nor underweight. Similar results were reported in previous studies on the effect of deworming on preschool children [25, 26]. There is some evidence that deworming can reduce stunting in under-five children. Studies have shown that deworming programs can lead to improved nutritional status, increased body weight and height, and increased cognitive development. Studies conducted in India [27], Peru [12], and Uganda [28] found statistically significant improvement of weight in children who were dewormed every 6 months compared with their counterparts. Likewise, another study found a significant increase in height and weight after deworming, which suggests positive effects of deworming in reducing the risks of stunting and underweight in children [29]. Moreover, previous studies found significant effects of deworming on reducing risks of stunting in children [9, 30]. However, the evidence is not definitive and further research is needed to determine the full impact of deworming on stunting in under-five children. While deworming alone is not enough to address the problem of stunting, it is an important intervention that can help to reduce the prevalence of stunting in children.

The study showed that children age and mother's age had association on health outcomes. Older children were less likely to suffer from poor health outcomes; likewise, children from older mothers had also lower risk of suffering from poor health outcomes. Children less than 2 years old are more vulnerable to poor nutritional health outcomes because their bodies are still developing and their immune systems are not as developed as older children [31]. In addition, their bodies are more sensitive to changes in nutrition because their developmental processes are still in flux. The reason for greater protection among children over 2 years up to 5 years might be their bodies are better able to absorb and metabolize nutrients. They have more developed digestive systems, and their organs, such as the liver, are better able to process and breakdown food. This allows them to absorb more of the nutrients from food and helps to prevent malnourishment.

Our study observed gender differences on the likelihood of suffering from poor health outcomes. Females had higher odds of suffering from poor health outcomes than males. This gender difference might be attributed due to biological factors as males tend to have higher levels of insulin-like growth factor-1 (IGF-1) than females, which is linked to an enhanced ability to take in nutrients from their diet [32]. In addition, males have been observed to have greater bone density than females [33], providing them with a shield from malnourishment. Moreover, males have been found to have a higher metabolic rate, allowing for more effective processing and absorption of nutrients.

The study also showed an association between socioeconomic status (wealth quantile, level of education, and type of place of residence) on health outcomes. However, there were disparities in how each of this determinants of deworming influence health outcomes. While rich wealth quartile and higher level of education reduces the odds ratio of children suffering from poor health outcomes, living in rural areas increases the likelihood of children suffering from poor health outcomes. Socioeconomic factors, such as poverty and income, have been shown [34] to have an impact on the effectiveness of deworming with nutrition health outcomes. Poor families may lack the resources to access deworming treatments and adequate nutrition, resulting in a higher prevalence of worm infections and subsequent health issues. Furthermore, environmental factors, such as access to clean water and sanitation, can also be influential [35]. Poor sanitation can facilitate the spread of worm infections, while clean water can help to reduce the risk of contamination and reinfection [35]. Access to healthcare and education is also a critical factor in successful deworming with nutrition health outcomes [36]; without proper education about the risks and effects of worm infections, people may not recognize the need for deworming treatments. In addition, dietary practices can also affect these outcomes; a balanced, nutritious diet can help to reduce the risk of worm infections, while an unhealthy diet can lead to an increase in these infections [37].

In 2001, the World Health Assembly (Resolution number: WHA54.19) requested all member states with intestinal worm infestations of more than 20% to deworm all under-five children once a year. In addition, countries with worm infestations of 50% or more were requested to pursue deworming of all children under five years old twice a year to eradicate or minimize the intensity of worms and the consequences of worm infestation [38]. In response, Tanzania implemented mass drug administration to all children between 12 and 59 months, including twice-yearly vitamin A supplementation [39]. However, due to the lack of drug category data in the Tanzania DHS, it was not possible to subdivide the sample into groups according to the type of deworming drug used and correlate them with the outcomes. Mebendazole 500 mg was the drug of choice during mass drug administration among children under five in Tanzania for simplicity during service delivery, recording, and reporting.

The selection of a nationwide representative sample provided good statistical power and a large generalization impact in this study. This study, however, is subject to several limitations. The survey's cross-sectional design limits the study's ability to find a cause-and-effect association. In addition, the responses given by mothers/caregivers regarding reporting deworming treatment to their children are sometimes based on their ability to recall and could be recall bias. Therefore, another large study to account for the observed constraints is strongly urged.

## 5. Conclusion

This study found strong association of deworming and anemia; dewormed children were less likely to be anemic than their counterparts. Therefore, regular monitoring and evaluation of the effectiveness and impacts of deworming programs are strongly urged to inform the government and decision-makers on how to continue or strengthen the program.

## Figures and Tables

**Table 1:** Social-demographic characteristics of study participants in association with deworming.

Variables	Frequency *N* (%)	Deworming *N* (%)		*P* values
		Yes	No	
*Gender*				0.061
Male	4034 (50.7)	1593 (42.1)	2193 (57.9)	
Female	3928 (49.3)	1484 (39.9)	2231 (60.1)	
*Age group*				0.03
12–23	2237 (28.1)	814 (38.1)	1320 (61.9)	
24–35	1925 (24.2)	775 (42.7)	1042 (57.3)	
36–47	1891 (23.8)	777 (43.4)	1013 (56.6)	
48–59	1909 (24.0)	711 (40.4)	1050 (59.6)	
*Wealth status*				<0.001
Poor	3578 (44.9)	1018 (30.1)	2363 (69.9)	
Normal	1549 (19.5)	621 (42.1)	854 (57.9)	
Rich	2834 (35.6)	1438 (54.4)	1207 (45.6)	
*Mother's education*				<0.001
No education	1703 (21.4)	408 (25.2)	1213 (74.8)	
Primary education	5177 (65)	2044 (42.1)	2811 (57.9)	
Secondary and higher education	1082 (13.6)	625 (61)	400 (39)	
*Source of water*				<0.001
Improved	4407 (55.3)	1923 (46.6)	2202 (53.4)	
Unimproved	3555 (44.7)	1154 (34.2)	2222 (65.8)	
*Toilet facilities*				<0.001
Improved	2127 (26.7)	1124 (57.3)	836 (42.7)	
Unimproved	5835 (73.3)	468 (35.9)	835 (64.1)	
*Mother's age*				0.02
Less than 30 years	4648 (58.4)	1730 (39.5)	2646 (60.5)	
More than 30 years	3314 (41.6)	1347 (43.1)	1778 (56.9)	

**Table 2:** Prevalence of underweight, stunting, and anemia and association with deworming in children.

Health outcome		Frequency *N* (%)	Deworming *N* (%)		*P* values
			Yes	No	
*Stunting*					0.001
No	4152 (60.5)		1788 (43.1)	2360 (56.9)	
Yes	2711 (39.5)		1057 (39)	1653 (61)	
*Underweight*					0.004
No	5842 (85)		2463 (42.2)	3371 (57.8)	
Yes	1033 (15)		387 (37.4)	647 (62.6)	
*Anemia*					<0.001
No	3029 (44.2)		1381 (45.6)	1646 (54.4)	
Yes	3823 (55.8)		1460 (38.25)	2358 (61.8)	

**Table 3:** Multilevel mixed effect logistic regression analysis of determinants of deworming on health outcomes (stunting, anemia, and underweight) among children aged 12–59 month in Tanzania.

Variables	Categories	Model I		Model II		Model III	
		AOR	95% CI	AOR	95% CI	AOR	95% CI
Child age (months)	12–23	1					
	24–35	0.89	[0.82–0.97]^*∗∗∗*^			0.89	[0.82–0.97]^*∗∗∗*^
	36–47	0.88	[0.81–0.96]^*∗∗∗*^			0.88	[0.81–0.96]^*∗∗∗*^
	48–59	0.94	[0.86–1.02]			0.94	[0.86–1.02]

Sex of child	Male	1					
	Female	1.01	[0.95–1.07]				

Mothers age (years)	15–24	1.00					
	25–34	0.89	[0.81–0.97]^*∗∗∗*^			0.89	[0.81–0.97]^*∗∗∗*^
	35–49	0.88	[0.79–0.99]^*∗∗*^			0.88	[0.79–0.98]^*∗∗*^

Mothers education level	No education	1					
	Primary education	0.81	[0.75–0.88]^*∗∗∗*^			0.83	[0.77–0.91]^*∗∗∗*^
	Higher education	0.58	[0.49–0.68]^*∗∗∗*^			0.61	[0.52–0.72]^*∗∗∗*^

Type of toilet facility	Improved	1					
	Unimproved	1.38	[1.25–1.52]^*∗∗∗*^			1.18	[1.02–1.37]^*∗∗*^
	Constant	0.67	[0.59–0.77]^*∗∗∗*^				

Wealth quintile	Poor			1			
	Neutral			0.84	[0.78–0.91]^*∗∗∗*^	0.90	[0.80–1.01]^*∗*^
	Rich			0.69	[0.62–0.76]^*∗∗∗*^	0.81	[0.70–0.93]^*∗∗∗*^

Type of place of residence	Urban			1			
	Rural			1.02	[0.91–1.14]		

Water source	Improved			1			
	Unimproved			1.08	[1.01–1.16]^*∗∗*^	1.01	[0.92–1.11]

^*∗∗∗*^ *p* < 0.01, ^*∗∗*^*p* < 0.05, ^*∗∗*^*p* < 0.1. AOR: adjusted odds ratio. Model I adjusted for individual-level characteristics, model II adjusted for community-level characteristics, model III adjusted for both individual- and community-level characteristics. Significant variables in model I and II were used forward for model III.

## Data Availability

To access TDHS 2015 dataset, you must register in the repository of the DHS Program and make a request.

## Abbreviations

HAZ: Height for age *Z*-score
STH: Soil-transmitted helminths
TDHS: Tanzania Demographic and Health Survey
WAZ: Weight for age *Z*-score.
